# Dual Polarization 2 × 2 Array Ku-Band Antenna with Improved Polarization Purity

**DOI:** 10.3390/s26082435

**Published:** 2026-04-15

**Authors:** Tae-Hak Lee, Hyungseok Nam, Jungwon Seo, Sangyoon Lee, Kwonki Hong, Seongmin Pyo

**Affiliations:** 1 Department of Electrical and Electronic Engineering, Yuhan University, Bucheon 14780, Republic of Korea; 2 Department of Datalink 2 Team, Hanhwa Systems Co., Ltd., Seongnam-si 13524, Republic of Korea; hyungseok.nam@hanwha.com (H.N.); jungwon81.seo@hanwha.com (J.S.); hongsboy.hong@hanwha.com (K.H.); 3 Department of Information and Communication Engineering, Hanbat National University, Daejeon 34158, Republic of Korea

**Keywords:** dual-polarization, linear polarization, microstrip, array antenna, Ku-band, power divider, cross-polarization

## Abstract

This letter presents a Ku-band 2 × 2 patch array antenna that supports dual-polarization operation using a simple cooperative feed network. Depending on the selected input port of the proposed simple feed network, the 2 × 2 array antenna radiates either vertically or horizontally polarized waves. The proposed feed structure consists of two serially connected power dividers placed on the same geometrical plane, enabling dual-polarization without additional multilayer routing. The microstrip line-based feed network also enables a 180° reversed placement of the radiating elements thereby improving the cross-polarization ratio of the proposed array antenna, achieving better than 30 dB across the operating band. The fabricated antenna, designed for a center frequency of 14.9 GHz with a 6.8% fractional bandwidth, demonstrates a realized gain higher than 10 dB for both polarization modes. Measurement results in terms of the input impedance bandwidth, isolation, gain, and cross-polarization ratio are in good agreement with simulation results.

## 1. Introduction

In next-generation wireless systems such as high-speed data communication, low Earth orbit (LEO) satellite constellations, and 5G networks, the need for multiple linear-polarization antennas has become increasingly important to improve signal quality, suppress interference, and enhance spectrum utilization [1,2,3]. Various antenna technologies, including waveguide-based structures, microstrip patches, three-dimensional helical antennas, and dielectric resonators with or without metasurfaces, have been reported for these applications [4,5,6,7,8,9]. Among these, microstrip patch arrays are particularly attractive due to their ease of implementation, low cost, and suitability for mass production. Recent studies have also emphasized the importance of achieving high polarization purity and low cross-polarization levels in dual-polarized antenna systems for sensing and radar applications [10,11]. Recent studies have reported various dual-polarized patch and array antennas with high isolation, wide bandwidth, and improved polarization purity, including aperture-coupled stacked patch arrays, magnetically coupled patch arrays, and differentially driven patch antennas. Although these approaches achieve excellent electromagnetic performance, many of them still rely on multilayer or additional feed-control structures, which can increase fabrication complexity. For Ku-band terminal and SATCOM applications, compact dual-polarized antenna architectures with high polarization discrimination remain highly desirable [12,13,14,15]. In addition, differential feeding schemes and decoupling networks have been introduced to suppress undesired coupling between orthogonal polarization ports and to improve cross-polarization performance [16,17]. Although these approaches can achieve excellent polarization characteristics, they often rely on complex multilayer structures, additional feeding circuits, or precise phase-control mechanisms, which increase fabrication complexity and limit their applicability to compact and low-cost antenna systems. In particular, achieving both high polarization purity and structural simplicity remains a key challenge in compact dual-polarized array design. In [18], a microstrip-based array antenna demonstrated beam-steering capability using external power divider circuits for mmWave 5G applications. These studies indicate that achieving proper impedance matching, polarization balance, and cross-polarization suppression within a compact array structure remains a significant design challenge. In addition, recent Ku-band satellite terminal studies report that polarization purity exceeding 30 dB is often required to mitigate cross-polarization interference in dual-polarized links, with those required values demonstrated in practical receive chains for compact user terminals [19,20]. These requirements highlight the importance of simple and planar array architectures capable of maintaining high polarization discrimination without multilayer or three-dimensional feeding structures. However, many dual-polarized microstrip arrays still rely on multilayer feed routing or additional phase-control components, increasing fabrication complexity and limiting practical compactness. Such configurations also make it difficult to maintain balanced excitation and low cross-polarization when two orthogonal polarization ports share the same plane.

Motivated by these limitations, this work proposes a simple planar feed line concept that enables equal-phase or 180° reversed excitation through physical line-length adjustments without altering the electrical behavior of the power divider. This approach provides a highly manufacturable solution for compact Ku-band dual-polarized arrays. To address these challenges, this letter presents a Ku-band 2 × 2 microstrip patch array employing a simplified feed line configuration capable of supporting vertical and horizontal polarization using two input ports located on the same geometrical plane. By adjusting only the physical lengths of selected feed line segments while maintaining their electrical characteristics such as isolation at the design frequency, the proposed network enables 180° reversed placement of the radiating elements, thereby improving cross-polarization performance. In the following sections, the detailed design methodology of the proposed feed-line configuration is first described, followed by the implementation of the dual-polarized 2 × 2 patch array. The operating principle of the equal-phase and 180° reversed-phase excitations is analytically explained, and the corresponding simulation results are presented. Finally, the fabricated prototype and measured results are provided to verify the effectiveness of the proposed approach.

## 2. Dual-Polarization Antenna Design

**Figure 1:**
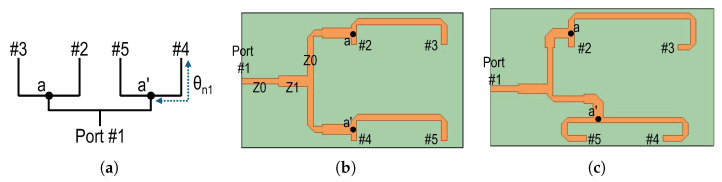
(**a**) Feed line schematic, (**b**) feed line design for equal phase, (**c**) feed line design for 180° reversed phase. w_1_ = 0.78 mm, w_2_ = 1.29 mm, l_*ch*_ = 0.9 mm.

Figure 1 shows the configuration of the proposed feeding structure for realizing dual-polarization operation. A conventional cooperative feed network is employed for the 2 × 2 array, where two 1:2 power dividers are connected in series and two nodes, a and a′, are defined as shown in Figure 1a. Note that although the schematic represents the feed lines with uniform solid lines, the line width can be optimized to maintain the intended power-splitting ratio. In the proposed design, the line Z_1_ in Figure 1b is set to 35.4 Ω on a 10-mil-thick substrate with a dielectric constant of 2.2 to achieve the required 1:2 split. The detailed width and chamfering dimension of the microstrip line are given in the caption of Figure 1. The electrically equivalent positions of the nodes are also depicted in Figure 1b,c, showing that a 50 Ω microstrip line connects each node to the output ports of the divider. This indicates that an additional design degree of freedom exists, allowing adjustment of the physical line length without changing the characteristics of the power divider.

**Figure 2:**
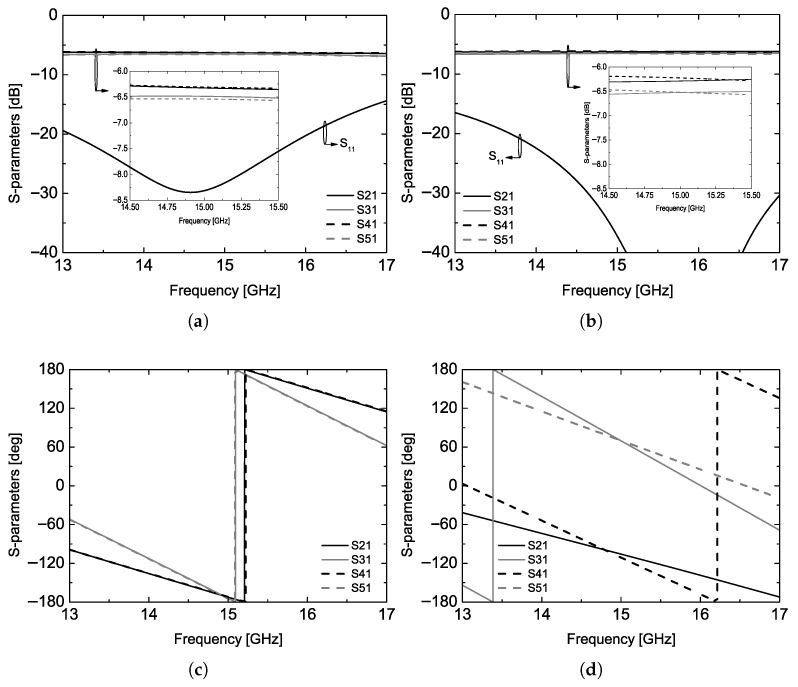
Simulation results of the feed line configurations: (**a**) S-parameters magnitude of the equal output phase design, (**b**) S-parameters magnitude of the reverse output phase design, (**c**) S-parameters phase of the equal output phase design, (**d**) S-parameters phase of the reverse output phase design.

In the proposed dual-polarization antenna, the physical length of the 50 Ω microstrip line from each node to its output ports is adjusted while maintaining the electrical characteristics of the feed line at the center frequency. This asymmetric modification provides greater placement flexibility for the input ports, thereby preventing unwanted routing interference between the two feed paths placed in the same plane for dual-polarization operation. The simulated S-parameters of the feed line generating the equal output phase are shown in Figure 2. Please note that all key dimensions for the feed line design are given in the caption of Figure 1, so one can repeat the same transmission characteristic shown in Figure 2. An additional insertion loss of approximately 0.25 dB occurs in the longer transmission line section, while the input matching remains better than –20 dB over the 14.5–15.5 GHz band of interest. It is also noted that the transmission phase responses at the input and output ports coincide at the center frequency of 14.9 GHz, as shown in Figure 2c. The electrical lengths of the feed lines for vertical polarization are summarized in Table 1. The parameter α can be optimized during the antenna design; in this work, α was set to approximately –170° considering the electrical spacing between the radiating patches.

The operating principle of the proposed feed-line configuration can be further understood based on the transmission-line phase relation. For a lossless microstrip line, the transmission phase at each output port can be expressed as

(1) $$\theta_{n1}\left(f\right)=\beta_{0}\left(f\right)l_{n},$$

where $\beta_{0}$(*f*) denotes the guided phase constant of the 50-Ω microstrip line at frequency *f*, and *l*_*n*_ represents the physical length from the reference node to the n-th output port. It should be noted that $\beta_{0}$ is the propagation-related phase constant of the transmission line and is distinct from the design parameter β defined in Table 1, where β represents a reference electrical-length parameter used to specify the required phase distribution in the reversed-phase configuration.

In the equal-phase design, the line lengths are selected such that

(2) $$\theta_{21}=\theta_{31}=\theta_{41}=\theta_{51}$$

at the center frequency. Since the characteristic impedance of each branch remains 50 Ω and the impedance transformation condition of the 1:2 power divider is preserved, modifying the physical line length does not alter the intrinsic power-splitting ratio or input matching characteristics at the design frequency. From a network perspective, this corresponds to a reference-plane shift at each output port while maintaining the scattering parameters of the divider.

For the 180° reversed configuration, the line lengths are determined to satisfy

(3) $$\theta_{n1}-\theta_{m1}=180^{\circ }$$

for adjacent radiating elements at the center frequency. This alternating phase excitation modifies the aperture current distribution and effectively suppresses undesired orthogonal field components at boresight. As a result, the cross-polarization level can be reduced without introducing additional phase-shifting circuits or multilayer feed routing, thereby preserving the planar and manufacturable nature of the proposed array architecture.

In addition to the design that achieves the identical output phase at each port, as shown in Figure 1b, the proposed feed line design theory can also be applied when the radiating elements are placed in a 180° reversed configuration. For more details, the feed line structure which exhibits the 180° reverse transmission phase at each output port is shown in Figure 1c and its simulation results are also given in Figure 2b,d. As shown in the simulated phase results, the phase value at the output port changes sequentially by 180° in a clockwise direction. The electrical lengths are also listed in Table 1. As shown in the values for the reversed phase design, the physical length of the 50 Ω line from the node to output ports are different from each other and the slopes of phase response of the S-parameters shown in Figure 2d confirm the design results. The β value can also be optimized based on the space between radiating elements. The proposed simple feed line structure with asymmetrically elongated 50 Ω lines can allow two input ports for the different polarization operation to be placed on the same plane, so it makes the proposed dual-polarization antenna structure simple. In addition, the cross-polarization performance of the proposed array antenna is improved from using the feed line configurations shown in Figure 1b,c together, as the feed line configuration achieves unequal input phases and physical distance between radiating patches, simultaneously.

**Figure 3:**
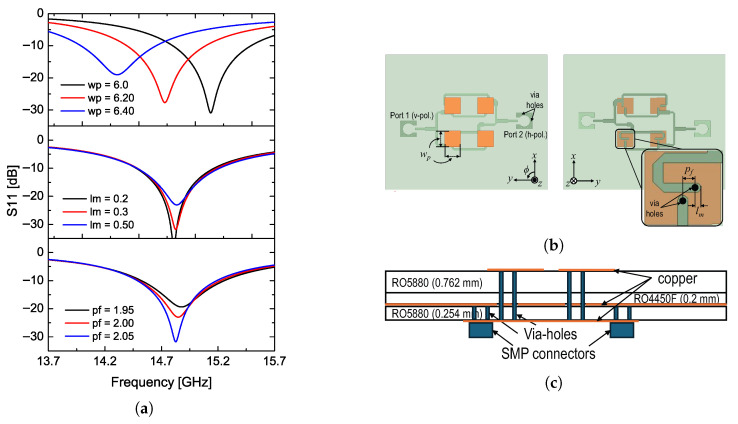
Proposed dual-polarization antenna design: (**a**) parameter study for impedance matching characteristic, (**b**) top and bottom view, (**c**) side view. ($w_{p}$ = 5.93, $p_{f}$ = 1.015, $l_{m}$ = 0.39, all in [mm]).

Figure 3 shows the circuit configuration of the proposed antenna which is designed based on the feed networks shown in Figure 2 along with 4 rectangular radiating patches. The single rectangular patch is optimized by adjusting both the feed position and the microstrip length from the feed point to the open end. The simulation results regarding each design parameter are given in Figure 3c. The design parameters, $p_{f}$ and $l_{m}$, are indicated in Figure 3a, and it should be noted that the radiating patch must be square to ensure comparable radiation performance for both polarization operations. The optimized design values are provided in the caption of Figure 3.

The feed line, which provides an equal input phase as shown in Figure 1b, is applied at port #1 for the vertical polarization. For generating the horizontally polarized wave, the feed line structure shown in Figure 1c is applied at port #2 to support the 180° reversed arrangement. The copper-plated via-holes with a diameter of 0.4 mm connect the feed line from the bottom layer to the top radiating patch, as shown in the side view of Figure 3b. A 0.2 mm-thick prepreg layer is inserted between two substrates: a 0.762 mm-thick layer for the radiating patch and a 0.254 mm-thick layer for the feed line. All design parameters, including the via-hole diameter and substrate thicknesses, are thoroughly optimized to achieve the required operational bandwidth centered at 14.9 GHz and isolation between ports better than −40 dB.

**Figure 4:**
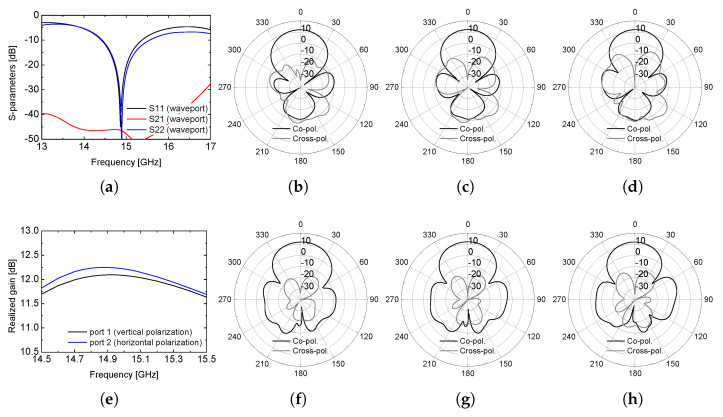
Simulation results: (**a**) S-parameters, (**b**) vertical polarization radiation pattern at 14.5 GHz, (**c**) radiation pattern at 14.9 GHz, (**d**) radiation pattern at 15.5 GHz, (**e**) realized gain, (**f**) horizontal polarization radiation pattern at 14.5 GHz, (**g**) radiation pattern at 14.9 GHz, (**h**) radiation pattern at 15.5 GHz.

The simulation results are shown in Figure 4, and were obtained using Ansys Electronics Desktop 2021 R2. The S-parameter results show that the proposed array antenna can demonstrate excellent impedance-matching performance at the center frequency of 14.9 GHz with the −10 dB S_11_ from 14.5 GHz to 15.5 GHz with the required isolation performance smaller than −30 dB. The simulated values of the realized gain for both polarization operation show higher than 11.5 dB. The radiation patterns at the center frequency, when each port is excited, are also provided. The simulated peak realized gain at 14.9 GHz is approximately 11.8 dB and 11.6 dB for the vertical and horizontal polarizations, respectively. The half-power beamwidth (HPBW) in the principal plane is approximately 38–42° for both polarization modes, indicating nearly symmetric radiation characteristics. In addition, the first sidelobe level is maintained below −13 dB at the center frequency, demonstrating stable array performance without excessive amplitude imbalance. The isolation between the two input ports remains better than −30 dB across the entire 14.5–15.5 GHz band, confirming that the planar feed configuration does not degrade inter-port decoupling. Note that the radiation patterns are plotted with a fixed ϕ = 0° for both polarizations at the center, lower, and upper frequencies because the dual-polarization patterns are symmetric. In other words, the radiation characteristics for the vertical polarization at ϕ = 90° are very similar to those shown in Figure 4f–h in terms of gain, beamwidth, and sidelobe level. It should be noted that the cross-polarization ratio at boresight exceeds 30 dB across the operating band as given in the radiation pattern results.

## 3. Verification

**Figure 5:**
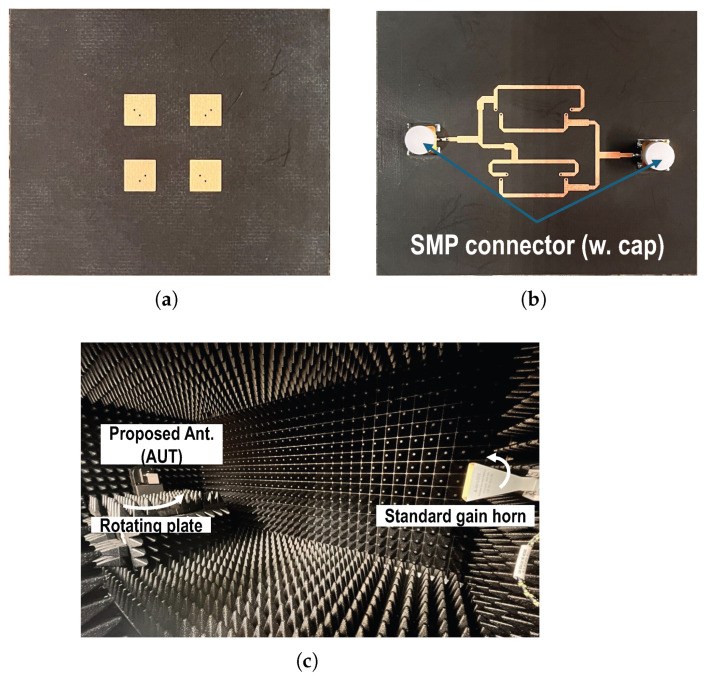
(**a**) Photograph of the fabricated antenna (top), (**b**) photograph of the fabricated antenna with SMP connectors (bottom), (**c**) radiation pattern measurement setup in an anechoic chamber.

**Figure 6:**
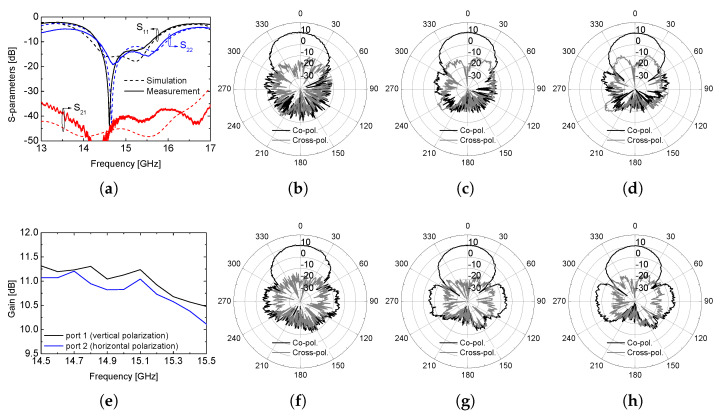
Measurement results: (**a**) S-parameters, (**b**) vertical polarization radiation pattern at 14.5 GHz, (**c**) radiation pattern at 14.9 GHz, (**d**) radiation pattern at 15.5 GHz, (**e**) peak gain, (**f**) horizontal polarization radiation pattern at 14.5 GHz, (**g**) radiation pattern at 14.9 GHz, (**h**) radiation pattern at 15.5 GHz.

The photographs of the fabricated antenna and the measurement setup in an anechoic chamber are shown in Figure 5. Two SMP connectors are soldered onto the feed line structure, as shown in Figure 5b, with their caps attached. The measured S-parameters are presented in Figure 6a, and non-ideal characteristics such as the dielectric loss associated with the commercial SMP connectors are included in the simulations using the CAD model provided by the manufacturer (Amphenol RF, Danbury, CT, USA, SMP-MSLD-PCS20T). The fabricated array antenna also achieves a −10 dB impedance bandwidth from 14.5 GHz to 15.5 GHz, with return loss better than −15 dB at the center frequency for both input ports. The measured isolation between the two ports remains better than 35 dB across the 14.5–15.5 GHz band, with a peak isolation exceeding 40 dB near the center frequency. This confirms that the asymmetrically routed feed network does not introduce significant coupling between the orthogonal polarization paths. The measured peak gain across the operating band is approximately 1 dB lower than the simulated value. The measured peak gain at the center frequency is approximately 10.8 dB for the vertical polarization and 10.6 dB for the horizontal polarization. The measured half-power beamwidth is approximately 40° in the principal plane, showing good agreement with the simulated results. The sidelobe level is observed to be below −12 dB across the operating band, which is consistent with the predicted array behavior. These discrepancies are mainly attributed to additional conductor and dielectric losses introduced by the soldered SMP connectors, as well as fabrication tolerances in the prepreg thickness and via metallization. Nevertheless, the fabricated antenna still exhibits a gain higher than 10 dB, and the frequency-dependent gain variation follows a trend consistent with the simulated results for both polarization modes. The measured radiation patterns are shown in Figure 6c–h, and they agree well with the simulations in terms of beamwidth, sidelobe level, and cross-polarization ratio at boresight. The noticeable difference observed in the backward radiation region is primarily related to the practical measurement configuration. The rotating fixture and supporting structure beneath the antenna under test may not be consistent with the simulation environment, particularly at θ = 180°, where radiation from the bottom feed layer interacts with the mounting platform. Such effects mainly influence the backward radiation characteristics and have negligible impact on the forward gain and boresight cross-polarization performance, which are the primary figures of merit for the intended application.

**Figure 7:**
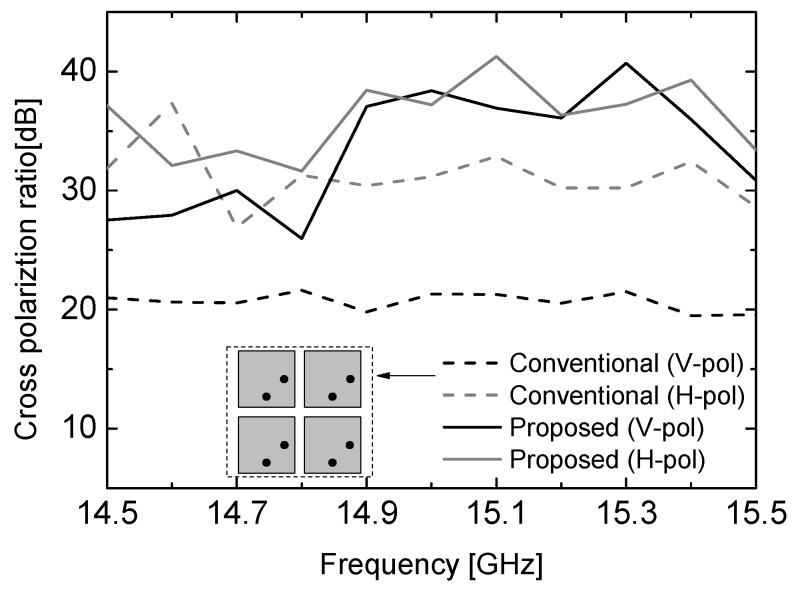
Measured cross polarization ratio of the proposed dual-polarization antenna.

The measured cross-polarization ratios of the proposed antenna and those of the conventional 2 × 2 array antenna are compared in Figure 7. The feed line of the conventional array is designed with the structure shown in Figure 1b for both input ports, and the corresponding feeding points of each radiating patch are indicated in the inset of Figure 7. The cross-polarization ratios for input port #1 (vertical polarization) and port #2 (horizontal polarization) are plotted using black and gray lines, respectively.

As observed, the proposed array employing the 180° reversed configuration provides improvements greater than 10 dB and 5 dB for the vertical and horizontal polarizations, respectively, at the center frequency of 14.9 GHz. This improvement results from the intentional phase alternation between adjacent radiating elements, which modifies the overall aperture field distribution and reduces the coherent generation of undesired orthogonal field components at boresight. In addition, the asymmetric feed-line routing introduces unequal physical spacing between radiating elements, further enhancing the polarization discrimination without requiring additional phase-shifting circuits or multilayer structures. These results confirm that the proposed antenna provides improved polarization purity while maintaining a simple and planar feed structure, making it suitable for compact wireless communication terminals. A comparison with previously reported dual-polarized antennas is summarized in Table 2. The proposed antenna achieves a cross-polarization ratio lower than −35 dB and port isolation higher than 40 dB, which are comparable to or better than those reported in [12,13,14,15,16]. In particular, while the antennas in [12,13] employ larger array configurations such as 4 × 4 and 8 × 8 arrays to achieve high gain and polarization performance, the proposed 2 × 2 array maintains a similar level of cross-polarization suppression with a significantly reduced footprint. Compared to directly fed or differentially fed structures in [16,17], the proposed antenna achieves competitive polarization purity without requiring additional multilayer routing or complex feeding networks. Note that the antennas compared in Table 2 have different array configurations and operating conditions. Therefore, the comparison is intended to provide a general performance reference rather than a direct one-to-one equivalence. The main contribution of this work lies in achieving high polarization purity using a simple planar feed configuration, which offers advantages in terms of structural simplicity, compactness, and ease of fabrication.

## 4. Conclusions

This study presents a Ku-band 2 × 2 dual-polarized microstrip patch array antenna employing a simple planar feed network. By adjusting only the physical lengths of selected 50-Ω microstrip line segments, the proposed structure enables equal-phase and 180° reversed-phase excitations on a single geometrical plane without requiring multilayer routing or additional phase-control circuits. The proposed configuration achieves a cross-polarization ratio exceeding 35 dB, port isolation better than 40 dB, and a realized gain higher than 10 dB across the operating band. Both simulation and measurement results show good agreement, confirming the validity of the proposed design approach. In addition, a parametric study on the radiating patch has been conducted to ensure proper impedance matching and to provide further insight into the design methodology, enhancing the reproducibility of the proposed antenna. Compared to conventional multilayer or differentially fed dual-polarized arrays, the proposed antenna offers a simpler structure while maintaining competitive polarization performance. Therefore, the proposed design provides an effective and practical solution for compact dual-polarized antenna systems in Ku-band wireless and satellite communication applications.

## Figures and Tables

**Table 1:** Feed line design parameters.

	$\theta_{21}$	$\theta_{31}$	$\theta_{41}$	$\theta_{51}$
equal phase	α	α + $\lambda_{0}$	α	α + $\lambda_{0}$
180° reverse phase	β	β + $1.5\lambda_{0}$	β + $\lambda_{0}$	β + $0.5\lambda_{0}$

**Table 2:** Comparison table.

	Antenna Type	Feeding Method	*f*_0_ [GHz]	Size [mm]	Gain [dB]	XPD (θ = 0)	Isolation [dB]
[12]	Patch (single)	Proximity coupling	3.5	∼120 × 120 × 10	∼9.3	∼−38	>50
[16]	Patch (single)	Direct feed	2.8	∼27 × 27 × 14	∼5.1	<−32	∼ 52
[17]	Patch (1 × 5)	Direct feed	2.45	∼220 × 40 (w.o. PD) *	6.5	<−34	∼ 51
[18]	Patch (4 × 4)	Coupled line coupling	28	37.2 × 37.2 × 1.2	17.37	∼−30	∼22.6
[19]	Patch (8 × 8)	Proximity coupling	19.5	∼80 × 80	28.5 (LP)	∼−30	>20
This work	Patch (2 × 2)	Direct feed	14.9	50 × 50 × 1.2	∼10.9	<−35	>40

* The 3D antenna size is not clearly reported when the antenna is measured with an external power divider.

## Data Availability

The data that support the finding of this study are available within the article.
